# All-cause mortality and cardiovascular events in a Spanish nonagenarian cohort according to type 2 diabetes mellitus status and established cardiovascular disease

**DOI:** 10.1186/s12877-022-02893-z

**Published:** 2022-03-18

**Authors:** MA Salinero-Fort, J. Mostaza, C. Lahoz, J. Cárdenas-Valladolid, J. I. Vicente-Díez, P. Gómez-Campelo, J. M. de Miguel-Yanes

**Affiliations:** 1 Fundación de Investigación e Innovación Biosanitaria de Atención Primaria, Madrid, Spain; 2 Instituto de Investigación Sanitaria del Hospital Universitario La Paz (IdIPAZ, Madrid, Spain; 3 Red de Investigación en Servicios de Salud en Enfermedades Crónicas (REDISSEC), Madrid, Spain; 4 Subdirección General de Investigación y Documentación, Consejería de Sanidad, Madrid, Spain; 5 Servicio de Medicina Interna, Hospital Universitario La Paz-Cantoblanco-Carlos III, Madrid, Spain; 6 Fundación de Investigación e Innovación Biosanitaria de Atención Primaria, Sistemas de Información, Madrid, Spain; 7 Universidad Alfonso X El Sabio, Madrid, Spain; 8 Centro de Salud Monóvar, Comunidad de Madrid Servicio Madrileño de Salud, Madrid, Spain; 9 Departamento de Medicina Interna, Hospital General Universitario Gregorio Marañón, Madrid, Spain; 10 Facultad de Medicina, Universidad Complutense de Madrid (UCM), Madrid, Spain; 11 Instituto de Investigación Sanitaria Gregorio Marañón (IiSGM), Madrid, Spain

**Keywords:** Nonagenarians, Diabetes mellitus, Cardiovascular disease, Mortality rates

## Abstract

**Background:**

Despite the progressive aging of the population in industrialized countries, few studies have focused on the natural history of cardiovascular disease in the very old, and recommendations on prevention of cardiovascular disease in this population are lacking. We aimed to analyze all-cause mortality and cardiovascular events according to prevalent type 2 diabetes mellitus and established cardiovascular disease in nonagenarians from a Mediterranean population.

**Methods:**

We analyzed the primary health records of all nonagenarians living in the Community of Madrid (*N* = 59,423) and collected data for 4 groups: Group 1, individuals without T2DM or established CVD (T2DM-, CVD-); Group 2, individuals without T2DM but with established CVD (T2DM-, CVD +); Group 3, individuals with T2DM but without established CVD (T2DM + , CVD-); and Group 4, individuals with both T2DM and established CVD (T2DM + , CVD +), taking into account the influence of sex on the outcomes. Follow-up was 2.5 years. The primary outcomes were cumulative incidence and incidence density rates for all-cause mortality, non-fatal myocardial infarction, non-fatal stroke (the first composite primary outcome [CPO1]), combined with heart failure (CPO2). We evaluated the adjusted effect of each group on all-cause mortality (Cox regression).

**Results:**

Mean age was 93.3 ± 2.8 years (74.2% women). Hypertension, dyslipidemia, heart failure, albuminuria, and estimated glomerular filtration rate < 60 mL/min/1.73 m2 were significantly more prevalent in G4 than in the other groups (all *p* values < 0.001). We observed significantly higher cumulative incidence rates for all-cause mortality, CPO1, and CPO2 in participants belonging to G4 (all *p* values ≤ 0.001). People in G2 presented higher rates of all-cause mortality, heart failure, CPO1, and CPO2 than people in G3 (all *p* values ≤ 0.001). In the fully adjusted model, G4 independently predicted all-cause mortality (HR = 1.48 [95% CI, 1.40 to 1.57] vs reference G1 [*p* < 0.01]). In addition, significant HRs were recorded for cardiovascular disease alone (G2) and type 2 diabetes mellitus alone (G3) (1.13 and 1.14, respectively; both *p* values < 0.01).

**Conclusions:**

In Spanish nonagenarians, established cardiovascular disease and type 2 diabetes mellitus conferred a modest risk of all-cause mortality. However, the simultaneous presence of both conditions conferred the highest risk of all-cause mortality.

**Supplementary Information:**

The online version contains supplementary material available at 10.1186/s12877-022-02893-z.

## Background

It is widely accepted that type 2 diabetes mellitus (T2DM) [1, 2] and established cardiovascular disease (CVD) [3, 4] increase the risk of morbidity and mortality. The burden of T2DM and established CVD with respect to incident cardiovascular events and mortality may differ according to sex, ethnic background, environmental conditions, or age. The Hoorn study, which was performed in Dutch patients aged 50 to 75 years, showed that women with T2DM and no history of CVD had a cardiovascular risk similar to that of women without T2DM and a history of CVD, whereas in men, the presence of established CVD conferred the highest cardiovascular risk [5]. In a recent Spanish study, which is illustrative of a Mediterranean country, people with T2DM had more than double the risk of ischemic stroke after adjusting for other risk factors, whilst T2DM was an independent factor for in-hospital mortality only in women [6].

More specifically, few studies have focused on the natural history of cardiovascular disease in the very old population, since frail older people are usually excluded from randomized controlled trials [7, 8]. Consequently, in the American Diabetes Association’s 2021 review of “Standards of Medical Care in Diabetes”, the chapter on diabetes treatment in older adults cites no single randomized controlled trial evaluating the impact of hypoglycemic treatment on cardiovascular morbidity and mortality in the elderly [9]. One rare exception is the HYVET study, which highlighted the beneficial impact of treating hypertension on incident cardiovascular disease in older persons [10].

Here, we aimed to compare the incidence of all-cause mortality and cardiovascular events among four subsets of Spanish nonagenarians: Group 1, individuals without T2DM or established CVD (T2DM-, CVD-); Group 2, individuals without T2DM but with established CVD (T2DM-, CVD +); Group 3, individuals with T2DM but without established CVD (T2DM + , CVD-); and Group 4, individuals with both T2DM and established CVD (T2DM + , CVD +), taking into account the influence of sex on the outcomes.

## Methods

### Study design

The study design has been previously published [11]. Briefly, we performed a population-based study in two phases: first, a cross-sectional study of all residents aged ≥ 90 years living in the Community of Madrid (Spain); second, a 2.5-year follow-up study of this population. The Community of Madrid is a public entity providing healthcare coverage to 100% of the population. It provides primary care through 3,881 general practitioners working in 265 health centres. All residents have an electronic clinical record in primary care. The record constitutes a clinical database of anonymised data. As at 31 December 2015, it contained data for 61,059 persons aged ≥ 90 years. Data were available for 59,423 subjects, of whom 11,645 (19.6%) had been diagnosed with T2DM.

### Variables and definitions

Data for all participants (*n* = 59,423) were collected through electronic clinical records in primary care, and included age, sex, cardiovascular risk factors, comorbidities and medication prescriptions as at 31 December 2015. Comorbidities were recorded according to the International Classification of Primary Care-Second Edition (ICPC-2).

Basic and instrumental activities of daily life were measured using the Barthel Index [12] and the Lawton and Brody Scale [13], respectively. As potential confounders, we collected information on the items included in the Charlson’s comorbidity index [14], which predicts 10-year mortality according to a wide range of comorbid conditions (i.e., age, dementia, chronic obstructive pulmonary disease (COPD), diabetes with or without organ damage, connective tissue disease, chronic kidney disease (CKD), AIDS, liver disease, malignancy, congestive heart failure, established acute myocardial infarction, peripheral vascular disease, transient ischemic attack or stroke, hemiplegia, and peptic ulcer disease). Dementia was identified by the P70 code of ICPC-2.

CVD was defined as a diagnosis of angina pectoris, myocardial infarction, stroke or peripheral artery disease in the electronic clinical record (ICPC-2 codes K74, K75, K76, K90 and K92). The mortality data including date of death was extracted from the SIP-CIBELES database (Community Information System of the Community of Madrid), which has records for the entire population of individuals in the Community of Madrid who hold a public health card (with the right to access public health care services). Only all-cause mortality was available with no information on the cause of death.

### Follow-up

All participants were followed-up during 30 months (2.5 years), a time period that was deemed sufficient for the development of events given the age of the population. We calculated the cumulative incidence and the density incidence rates dividing the number of incident cases by the number of person-years of follow-up (× 1,000) for the following variables: all-cause mortality, non-fatal myocardial infarction, non-fatal stroke, the first composite primary outcome (CPO1) -defined as all-cause mortality, non-fatal myocardial infarction and non-fatal stroke-, and the second composite primary outcome (CPO2) -defined as all-cause mortality, non-fatal myocardial infarction, non-fatal stroke and heart failure-, for both men and women with and without T2DM, stratified by the absence or presence of established CVD.

The quality of the primary care electronic clinical record has previously been validated for research purposes [15], and the database has been widely used to study the epidemiology of cardiovascular risk factors in the study population [16].

### Statistical analysis

Continuous variables are presented as mean and standard deviation (SD), and categorical variables as percentages. Categorical variables were compared using the chi-square test. Continuous variables were compared using the t-test. Analysis of variance (ANOVA) was conducted with the Dunnett's T3 or Scheffe’s post hoc comparison test, and was used to compare the prespecified groups according to prevalent T2DM and established CVD, respectively.

The cumulative incidence values are expressed as either a rate (events/person-years) or as a proportion (events/person). The confidence interval (CI) at 95% of the crude incidence rates were calculated using the exact Poisson formula. The adjusted effect of the groups on all-cause mortality was analyzed by the Cox regression model, and the corresponding hazard ratios (HRs), and their 95% CIs were calculated. The adjusting variables were: age, sex, chronic obstructive pulmonary disease, solid cancer, leukemia/lymphoma, chronic kidney disease, dementia, heart failure, deep vein thrombosis/pulmonary thromboembolism, and atrial fibrillation (these clinical diagnoses are part of the Charlson’s comorbidity index). Data were processed using SPSS for Windows, V.19.0 (IBM Corp., Armonk, New York, USA).

## Results

At baseline, the cohort consisted of 59,423 nonagenarians, with a mean age of 93.3 ± 2.8 years, of whom 74.2% were women. Group 1 (T2DM (-), CVD (-)) was comprised by 37,078 people (77.3% women), group 2 (T2DM (-), CVD ( +)) by 10,700 people (64.4% women), group 3 (T2DM ( +), CVD (-)) by 8,043 people (76.4% women), and group 4 (T2DM ( +), CVD ( +)) by 3,602 people (65.6% women) (Table 1 and Supplementary Table S1). In each group, women were older, more frequently lived in nursing homes, had lower rates of functional independence, higher blood pressure and LDL-cholesterol levels, higher prevalence of obesity, hypertension, dyslipidemia and impaired renal function, but lower smoking rates than men (Supplementary Table S1).

**Table 1:** Baseline characteristics of the population of nonagenarians included in the study, according to type 2 diabetes mellitus (T2DM) status, and the presence or absence of prior cardiovascular disease (CVD)

	**Group 1: No T2DM, no prior CVD** **(*n* = 37,078)**	**Group 2: No T2DM, prior CVD** **(*n* = 10,700)**	**Group 3: T2DM, no prior CVD** **(*n* = 8,043)**	**Group 4: T2DM, prior CVD** **(*n* = 3,602)**	***p* value**	***p* value** (for comparison between group 2 and group 3)	***p* value** (for comparison between group 3 and group 4)
**Age (years), mean (SD)**	93.3 (2.8)	93.3 (2.7)	92.9 (2.5)	92.9 (2.4)	< 0.001	< 0.001	0.581
**Living in nursing home, n/N (%)**	5,479/37,078 (14.8)	1,342/10,700 (12.5)	1,098/8,043 (13.7)	489/3,602 (13.6)	< 0.001	0.015	0.884
**Barthel: Functionally independent, n/N (%)**	1,591/13,779 (12.5)	451/4,865 (9.3)	411/3,747 (11.0)	151/1,884 (8.0)	< 0.001	0.009	< 0.001
**Lawton & Brody: Functionally independent, n/N (%)**	1,507/13,649 (11.0)	434/4,873 (8.9)	372/3,713 (10.0)	129/1,869 (6.9)	< 0.001	0.084	< 0.001
**Charlson's index, mean (SD)**	4.8 (1.1)	5.9 (1.3)	6.0 (1.2)	7.1 (1.4)	< 0.001	< 0.001	< 0.001
**Dementia, n/N (%)**	5,092/37,078 (13.7)	1,368/10,700 (12.8)	1,084/8,043 (13.5)	466/3,602 (12.9)	0.061	0.160	0.376
**COPD, n/N (%)**	3,244/37,078 (8.7)	1,259/10,700 (11.8)	803/8,043 (10.0)	413/3,602 (11.5)	< 0.001	< 0.001	0.016
**CKD, n/N (%)**	3,745/37,078 (10.1)	1,516/10,700 (14.2)	1,195/8,043 (14.9)	681/3,602 (18.9)	< 0.001	0.178	< 0.001
**Solid Cancer, n/N (%)**	2,379/37,078 (6.4)	789/10,700 (7.4)	606/8,043 (7.5)	315/3,602 (8.7)	< 0.001	0.796	0.028
**Leukemia/Lymphoma, n/N (%)**	137/37,078 (0.4)	36/10,700 (0.3)	31/8,043 (0.4)	10/3,602) (0.3)	0.781	0.251	0.398
**SBP (mmHg), mean (SD)**	130.3 (16.7)	128.6 (16.9)	131.5 (16.5)	130.2 (18.1)	< 0.001	< 0.001	0.015
**DBP (mmHg), mean (SD)**	70.7 (9.4)	69.7 (9.7)	70.5 (9.4)	69.2 (9.7)	< 0.001	< 0.001	< 0.001
**BMI > 30 kg/m**^**2**^**, n/N (%)**	2,597/13,048 (19.9)	815/4,140 (19.7)	907/3,764 (24.1)	333/1,617 (20.6)	< 0.001	< 0.001	0.005
**Current smoker, n/N (%)**	289/19,250 (1.5)	136/6,368 (2.1)	81/5,134 (1.6)	40/2,378 (1.7)	0.001	0.048	0.750
**Hypertension, n/N (%)**	25,010/37,078 (67.5)	7,936/10,700 (74.2)	6,505/8,043 (80.9)	2,978/3,602 (82.7)	< 0.001	< 0.001	0.020
**Dyslipidemia, n/N (%)**	11,903/37,078 (32.1)	4,452/10,700 (41.6)	3,640/8,043 (45.3)	1,860/3,602 (51.6)	< 0.001	< 0.001	< 0.010
**LDL-Cholesterol (mg/dl), mean (SD**)	109.5 (30.4)	97.4 (31.4)	98.6 (30.4)	88.8 (31.2)	< 0.001	0.155	< 0.001
**Chronic atrial fibrillation, n/N (%)**	5,322/37,078 (14.4)	2,485/10,700 (23.2)	1,424/8,043 (17.7)	846/3,602 (23.5)	< 0.001	< 0.001	0.041
**Heart failure, n/N (%)**	3,355/37,078 (9.0)	1,573/10,700 (14.7)	1,040/8,043 (12.9)	666/3,602 (18.5)	< 0.001	< 0.001	< 0.001
**Albuminuria, n/N (%)**	1,379/5,296 (26.0)	532/1,741 (30.6)	1,084/2,814 (38.5)	541/1,209 (44.7)	< 0.001	< 0.001	< 0.001
**eGFR < 60 mL/min/1.73 m**^**2**^**, n/N (%)**	6,128/10,300 (59.5)	2,103/3,369 (62.4)	1,770/2,787 (63.5)	880/1,307 (67.3)	< 0.001	0.373	0.017
**Antiaggregants, n/N (%)**	9,191/37,078 (24.8)	6,896/10,700 (64.4)	2,834/8,043 (35.2)	2,445/3,602 (67.9)	< 0.001	< 0.001	< 0.001
**Anticoagulants, n/N (%)**	6,163/37,078 (16.6)	2,708/10,700 (25.3)	1,589/8,043 (19.8)	947/3,602 (26.3)	< 0.001	< 0.001	< 0.001
**ACEI or ARB, n/N (%)**	11,940/37,078 (32.2)	4,530/10,700 (42.3)	3,371/8,043 (41.9)	1,764/3,602 (49.0)	< 0.001	0.580	< 0.001
**Statins, n/N (%)**	7,093/37,078 (19.1)	4,895/10,700 (45.7)	2,795/8,043 (34.8)	1,981/3,602 (55.0)	< 0.001	< 0.001	< 0.001

*COPD* Chronic Obstructive Pulmonary Disease, *CKD* Chronic Kidney Disease, *SBP* Systolic blood pressure, *DBP* Diastolic blood pressure, *BMI* Body mass index, *eGFR* estimated glomerular filtration rate, *ACEI* Angiotensin-converting enzyme inhibitors, *ARB* Angiotensin receptor blockers

Hypertension, dyslipidemia, heart failure, albuminuria and estimated glomerular filtration rate (eGFR) < 60 mL/min/1.73m^2^ were significantly more prevalent in group 4 (T2DM ( +), CVD ( +)) than in the other groups (all *p* values < 0.001). In addition, people in group 4 significantly more often received treatment with antiplatelet agents, anticoagulants, statins and angiotensin-converting enzyme inhibitors or angiotensin receptor blockers than people in the other groups (all *p* values < 0.001) (Table 1). People in group 3 (T2DM ( +), CVD (-)) had a higher prevalence of hypertension, dyslipidemia and albuminuria, and a lower prevalence of heart failure and atrial fibrillation than people in group 2 (T2DM (-), CVD ( +)) (all *p* values < 0.001) (Table 1).

We observed significantly higher rates of cumulative incidence of all-cause mortality, CPO1 and CPO2 in participants belonging to group 4 (all *p* values ≤ 0.001) (Table 2). People in group 2 presented higher rates of all-cause mortality, heart failure, CPO1 and CPO2 than people in group 3 (all *p* values ≤ 0.001), but a lower rate of incident acute myocardial infarction (*p* = 0.015), with no difference in the rate of stroke (*p* = 0.547) (Table 2).

**Table 2:** Cumulative incidence of all-cause mortality and cardiovascular events in the nonagenarian population of Madrid after 2 years of follow-up, according to type 2 diabetes mellitus (T2DM) status, and the presence or absence of prior cardiovascular disease (CVD)

	**Group 1: No T2DM, no prior CVD** **(*n* = 33,091)**	**Group 2: No T2DM, prior CVD** **(*n* = 9,991)**	**Group 3: T2DM, no prior CVD** **(*n* = 7,470)**	**Group 4: T2DM, prior CVD** **(*n* = 3,382)**	***p* value**	***p* value** (for comparison between group 2 and 3)	***p* value** (for comparison between group 3 and 4)
All-cause mortality, n (%)	9,183 (27.8)	3,385 (33.9)	2,318 (31.0)	1,360 (40.2)	< 0.001	< 0.001	< 0.001
Non-fatal AMI, n (%)	126 (0.4)	33 (0.3)	43 (0.6)	26 (0.8)	0.001	0.015	0.241
Non-fatal stroke, n (%)	903 (2.7)	321 (3.2)	228 (3.1)	132 (3.9)	< 0.001	0.547	0.022
Heart failure, n (%)	1,324 (4.0)	534 (5.3)	299 (4.0)	187 (5.5)	< 0.001	< 0.001	< 0.001
CPO1, n (%)	9,845 (29.8)	3,590 (35.9)	2,503 (33.5)	1,445 (42.7)	< 0.001	0.001	< 0.001
CPO2, n (%)	10,632 (32.1)	3,873 (38.8)	2,674 (35.8)	1,530 (45.2)	< 0.001	< 0.001	< 0.001

AMI: Acute myocardial infarction. CPO1: Composite primary outcome, number 1 (all-cause mortality, non-fatal myocardial infarction, non-fatal stroke). CPO2: Composite primary outcome, number 2 (all-cause mortality, non-fatal acute myocardial infarction, non-fatal stroke, heart failure)

Rates of all-cause mortality, CPO1 and CPO2 were higher in men than in women in every group (all *p* values < 0.05), but the differences found for CPO1 and CPO2 were mainly driven by all-cause mortality (Supplementary Table S2). Table 3 shows the density incidence for all the outcomes in each population group.

**Table 3:** Density incidence rates stratified by type 2 diabetes mellitus (T2DM) status, and presence or absence of prior cardiovascular disease (CVD)

	No T2DM, no prior CVD	No T2DM, prior CVD	T2DM, no prior CVD	T2DM, prior CVD
P-Y	62,147.3	18,107.7	13,663.2	5,892.8
Mortality rate per 1,000 P-Y (95% CI)	147.76 (144.74 – 150.78)	186.94 (180.64 – 193.23)	169.65 (162.75 – 176.56)	230.79 (218.53 – 243.06)
Non-fatal AMI rate per 1,000 P-Y (95% CI)	2.03 (1.67 – 2.38)	1.82 (1.20 – 2.44)	3.15 (2.21 – 4.09)	4.41 (2.72 – 6.11)
Non-fatal stroke rate per 1,000 P-Y (95% CI)	14.53 (13.58 – 15.48)	17.73 (15.79 – 19.67)	16.69 (14.52 – 18.85)	22.40 (18.58 – 26.22)
Heart failure rate per 1,000 P-Y (95% CI)	21.30 (20.16 – 22.45)	29.49 (26.99 – 31.99)	21.88 (19.40 – 24.36)	31.73 (27.19 – 36.28)
CPO1 rate per 1,000 P-Y (95% CI)	158.41 (155.28 – 161.54)	198.26 (191.77 – 204.74)	183.19 (176.02 – 190.37)	245.22 (232.57 – 257.86)
CPO2 rate per 1,000 P-Y (95% CI)	171.08 (167.83 – 174.33)	213.89 (207.15 – 220.62)	195.71 (188.29 – 203.13)	259.64 (246.63 – 272.65)

*P-Y* Person-years, *95% CI* 95% confidence interval, *AMI* Acute myocardial infarction, *CPO1* Composite primary outcome, number 1 (all-cause mortality, non-fatal myocardial infarction, non-fatal stroke), CPO2: Composite primary outcome, number 2 (all-cause mortality, non-fatal acute myocardial infarction, non-fatal stroke, heart failure)

After adjusting for age, sex, chronic obstructive pulmonary disease, solid cancer, leukemia/lymphoma, chronic kidney disease, dementia, heart failure, deep vein thrombosis/pulmonary thromboembolism, and atrial fibrillation, the presence of both prevalent T2DM plus established CVD (group 4) independently predicted all-cause mortality (HR = 1.48 (95% CI, 1.40 to 1.57), as compared to the absence of both T2DM and established CVD (reference group 1) (*p* value < 0.01). CVD alone (group 2) and T2DM alone (group 3) also predicted all-cause mortality, showing similar HRs (1.13 and 1.14, respectively), which were lower than the HR that we are reporting for group 4 (Table 4).

**Table 4:** Adjusted effect on all-cause mortality of the different categories according to type 2 diabetes mellitus (T2DM) status, and presence or absence of prior cardiovascular disease

	**HR**	**CI 95%**	***p* value**
Age	1.10	1.09–1.11	< 0.01
Male Gender	1.23	1.19–1.28	< 0.01
Group 1: T2DM (-) & CVD (-)	1		
Group 2: T2DM (-) & CVD ( +)	1.13	1.09–1.18	< 0.01
Group 3: T2DM ( +) & CVD (-)	1.14	1.09–1.20	< 0.01
Group 4: T2DM ( +) & CVD ( +)	1.48	1.40–1.57	< 0.01

Adjusted by history of Chronic obstructive pulmonary disease, solid cancer, leukemia/lymphoma, chronic kidney disease, dementia, heart failure, deep vein thrombosis or pulmonary thromboembolism, and atrial fibrillation

Finally, we performed a sensitivity analysis that included new adjustment factors such as systolic blood pressure, LDL cholesterol, statins, ACEI or ARB, and antiplatelet agents. The final model excluded 55% of the sample due to missing data and yielded very similar HRs for the four groups. Therefore, we preferred to maintain the least adjusted model.

In contrast to mortality (16,246 cases), the incidence of myocardial infarction (228 cases) and stroke (1,584 cases) was low, with the result that the confidence intervals, especially in myocardial infarction, were wide, and statistical significance was not reached in group 2 for myocardial infarction or in group 3 for stroke. However, group 4 showed a more prominent and significant effect on the incidence of stroke (HR = 2 [95%CI, 1.31 to 3.08]) and myocardial infarction (HR = 1.45 [95%CI, 1.20 to 1.74]) compared with the other groups. Group 2, on the other hand, showed an effect size similar to that of group 3 for the incidence of stroke (HR = 1.16 and 1.12, respectively). (Supplementary Tables 3 and 4).

## Discussion

Here we observed significantly higher rates of cumulative incidence of all-cause mortality in participants with T2DM and established CVD. At the time of follow-up, participants with established CVD without T2DM presented higher rates of all-cause mortality and heart failure than people with T2DM with no established CVD, albeit a lower rate of non-fatal acute myocardial infarction. Men significantly presented higher rates of all-cause mortality in each group, but we found no sex differences for incident non-fatal stroke or non-fatal myocardial infarction. In the model adjusted for age, sex, and the remaining clinical diagnoses included in the Charlson’s comorbidity index, prevalent T2DM plus established CVD (group 4) independently predicted all-cause mortality as compared to the absence of both T2DM and established CVD, with a higher HR than either T2DM or established CVD taken separately.

When specifically analyzing cardiovascular disease in the very elderly, several distinctive characteristics of this population must be taken into consideration. First, there may be differences in the prevalence of traditional risk factors when compared with younger populations, for example, hypertension, obesity, and cholesterol [17]. Second, the impact of traditional cardiovascular risk factors on cardiovascular outcomes might decline with aging, whereas the importance of other factors such as frailty or inflammatory conditions as conditions that predispose to cardiovascular events may rise [18]. And third, the clinical approach to the very elderly may differ according to cultural or social disparities between countries, for instance, with respect to the deprescription rate in frail or highly dependent aged people [19].

It did not come as a surprise that in our study having both T2DM and established CVD conferred the highest risk of suffering cardiovascular events and all-cause death during follow-up. All-cause mortality risk was lower when either T2DM or established CVD were present in the absence of the other condition. Established cardiovascular disease increases the risk of repeated cardiovascular events and prevalent T2DM further increases that risk [20]. The VALIANT trial showed that both previously known diabetes and incident diabetes increased the risk of new cardiovascular events and death after an acute myocardial infarction [21]. Other authors have reported an even higher impact on behalf of T2DM on the prognoses of the elderly patients after a myocardial infarction than in younger patients [22]. Moreover, the increased mortality risk conferred by T2DM has also been described for stroke [23]. From a practical standpoint, patients who have had a cardiovascular event are categorized as having the highest cardiovascular risk, but this risk seems to be even higher if T2DM is present, both in the short and the long-term after the cardiovascular event [24, 25].

We noticed that nonagenarians with established CVD lacking the diagnosis of T2DM presented higher crude rates of all-cause mortality than people with T2DM without established CVD. There has been a long-standing debate in the medical literature about whether prevalent T2DM and established coronary heart disease could be considered as “risk equivalent” for the development of cardiovascular events: some authors have defended this notion [26, 27], whilst others have not [28]. Additional research work brings diabetes at the same risk level as established cardiovascular disease but only for people with severe diabetes [29], or just for those with early-onset diabetes [30]. In the elderly, comorbidity and specific social and medical needs may make the comparison between both conditions to predict incident cardiovascular disease or mortality even less clear, since traditional risk factors lose weight at the same time that other conditions emerge to influence cardiovascular risk [31, 32]. As a consequence, specific cardiovascular risk prediction tools for the elderly have been put in place [33, 34]. Nevertheless, additional previous research has supported the idea of comparable cardiovascular risk between T2DM and established cardiovascular disease in the elderly [35]. In our study we found a similar all-cause mortality risk for both entities separately when we accounted for potential confounding variables.

We found no sex differences for incident non-fatal stroke or acute myocardial infarction, whereas all-cause mortality rate over time was higher in men. In a report from the TECOS study, even though women had worse cardiovascular risk factor profiles and were less often treated with indicated medications, they presented lower rates of cardiovascular events, which suggested that the cardioprotective effects of female sex extended to populations with T2DM [36]. On the opposite direction, other studies have found that the excess risk of acute coronary syndrome associated with T2DM is higher in women than in men [37]. Recent research in the Northern European population points to higher absolute rates of cardiovascular complications in men, whereas the relative rates of diabetes-related cardiovascular complications are higher in women than in men [38]. However, none of these studies focused on the very elderly, and methodological differences and distinct ethnic backgrounds may underlie the apparently discordant results.

We should point out some limitations of our study: some data were not available for a number of patients due to the fact that it was carried out under real conditions of clinical care. Thus, a possible bias due to missing data cannot completely be ruled out. However, recruitment bias was not an issue in our study, since we included virtually all the nonagenarians of our region. Also, we had no access to the cause of death of the participants; therefore, we designed modified variables for the combined endpoint “major cardiovascular events and mortality” -in our study, all-cause mortality instead of cardiovascular mortality was the outcome of interest.

## Conclusions

We found that in the Mediterranean population, prevalent T2DM plus established CVD independently predicted all-cause mortality as compared to the absence of both T2DM and established CVD. Established CVD and T2DM alone conferred similar mortality risks when they were present separately. These risks were of lower magnitude than the risk observed when both conditions were present. People with established CVD without T2DM presented higher rates of all-cause mortality and heart failure than people with T2DM without established CVD, but a lower rate of non-fatal acute myocardial infarction. While men presented significantly higher rates of all-cause mortality in each group, we found no sex differences for incident non-fatal stroke or non-fatal acute myocardial infarction. Additional research is needed to fully understand the impact of cardiovascular risk factors and the most suitable approach to their clinical management in the very elderly in both Mediterranean and non-Mediterranean populations.

## Supplementary Information

**Additional file 1.**

**Additional file 2.**

**Additional file 3.**

**Additional file 4.**

## Data Availability

The datasets used and/or analysed during the current study are available from the corresponding author on reasonable request.

## Abbreviations

T2DM: Type 2 diabetes mellitus
CVD: Cardiovascular disease
COPD: Chronic obstructive pulmonary disease
CKD: Chronic kidney disease
CPO1: First composite primary outcome
CPO2: Second composite primary outcome
SD: Standard deviation
ANOVA: Analysis of variance
CI: Confidence interval
