# Supplementary material for: All-cause mortality and cardiovascular events in a Spanish nonagenarian cohort according to type 2 diabetes mellitus status and established cardiovascular disease
Source: BMC Geriatr. 2022 Mar 18;22:224. doi: 10.1186/s12877-022-02893-z (PMC8931574; doi:10.1186/s12877-022-02893-z)
Supplement: Supplementary file 1 — Additional file 1. [file 12877_2022_2893_MOESM1_ESM.docx]

**Supplementary Table S1. Baseline characteristics of the population of nonagenarians included in the study, according to gender, type 2 diabetes mellitus (T2DM) status, and the presence or absence of prior cardiovascular disease (CVD).**

|  | **No T2DM, no prior CVD**  **(n= 37,078)** | | **No T2DM, prior CVD**  **(n= 10,700)** | | **T2DM, no prior CVD**  **(n= 8,043)** | | **T2DM, prior CVD**  **(n= 3,602)** | |
| --- | --- | --- | --- | --- | --- | --- | --- | --- |
|  | **Men**  **(n= 8,408)** | **Women**  **(n= 28,670)** | **Men**  **(n= 3,809)** | **Women (n= 6,891)** | **Men**  **(n= 1,899)** | **Women**  **(n= 6,144)** | **Men**  **(n= 1,239)** | **Women**  **(n= 2.363)** |
| **Age (years), mean (SD)** | 92.9 (2.5) | 93.4 (2.8)* | 92.9 (2.4) | 93.6 (2.8)* | 92.6 (2.2) | 93.1 (2.6)* | 92.5 (2.2) | 93 (2.5)* |
| **Living in nursing home, n/N (%)** | 891/8,408 (10.6) | 4,588/28,670 (16)* | 359/3,809 (9.4) | 983/6,891 (14.3)* | 168/1,899 (8.8) | 930/6,144 (15.1)* | 118/1,239 (9.5) | 371/2,363 (15.7)* |
| **Barthel: Functionally independent, n/N (%)** | 605/2,997 (20.2)* | 986/10,782 (9.1) | 263/1,644 (16)* | 188/3,221 (5.8) | 152/849 (17.9)* | 259/2,898 (8.9) | 86/639 (13.5)* | 65/1,245 (5.2) |
| **Charlson's comorbidity index, mean (SD)** | 5.9 (1.16)* | 5.8 (1.06) | 7.0 (1.4)* | 6.8 (1.2) | 7.1 (1.2) | 7.0 (1.2) | 8.3 (1.6)* | 8.0 (1.3) |
| **SBP (mmHg), mean (SD)** | 128.7  (16.3) | 130.7  (16.7)* | 127.2 (16.5) | 129.4  (17.0)* | 129.4 (15.8) | 132.1 (16.7)* | 129.3 (17.9) | 130.7 (18.2)* |
| **DBP (mmHg), mean (SD)** | 69.8 (9.4) | 71.0 (9.3)* | 68.7 (9.6) | 70.3 (9.8)* | 69.3 (9.3) | 70.9 (9.4)* | 68.3 (9.7) | 69.7 (9.7)* |
| **BMI > 30 kg/m^2^, n/N (%)** | 536/3,373  (15.9) | 2,061/9,675 (21.3)* | 285/1,694 (16.8) | 530/2,446 (21.7)* | 173/1,016 (17.0) | 734/2,748 (26.7)* | 106/646 (16.4) | 227/971 (23.4)* |
| **Current smoker, n/N (%)** | 160/4,523 (3.5)* | 129/14,727 (0.9) | 91/2,346 (3.9)* | 45/4,022 (1.1) | 52/1,268 (4.1)* | 29/3,866 (0.8) | 33/856 (3.9)* | 7/1,522 (0.5) |
| **Hypertension, n/N (%)** | 4,915/8,408 (58.5) | 20,095/28,670 (70.1)* | 2,487/3,809 (65.3) | 5,449/6,891 (79.1)* | 1,330/1,899 (70.0) | 5,175/6,144 (84.2)* | 949/1,239 (76.6) | 2,029/2,363 (85.9)* |
| **Dyslipidemia, n/N (%)** | 1,966/8,408 (23.4) | 9,937/28,670 (34.7)* | 1,385/3,809 (36.4) | 3,067/6,891 (44.5)* | 703/1,899 (37.0) | 2,937/6,144 (47.8)* | 590/1,239 (47.6) | 1,270/2,363 (53.7)* |
| **LDL-Cholesterol (mg/dl), mean (SD**) | 103.7  (28.8) | 111.1  (30.7)* | 90.0  (28.4) | 101.4 (32.2)* | 92.4  (27.5) | 100.5  (31.0)* | 81.8  (27.4) | 92.4  (32.5)* |
| **Chronic atrial fibrillation, n/N (%)** | 1,387/8,408 (16.5)* | 3,935/28,670  (13.7) | 880/3,809  (23.1) | 1,605/6,891 (23.3) | 372/1,899 (19.6)* | 1,052/6,144  (17.1) | 287/1,239 (23.2) | 559/2,363 (23.7) |
| **Heart failure, n/N (%)** | 662/8,408 (7.9) | 2,693/28,670 (9.4)* | 474/3,809  (12.4) | 1,099/6,891 (15.9)* | 222/1,899 (11.7) | 818/6,144 (13.3) | 218/1,239 (17.6) | 448/2,363 (19.0) |
| **Albuminuria, n/N (%)** | 404/1,258 (32.1)* | 975/4,038 (24.1) | 229/713 (32.1) | 303/1,028 (29.5) | 314/689 (45.6)* | 770/2,125 (36.2) | 226/464 (48.7)* | 315/745 (42.3) |
| **eGFR <60 mL/min/1.73 m^2^, n/N (%)** | 1,069/2,076 (51.5) | 5,059/8,224 (61.5)* | 645/1,133 (56.9) | 1,458/2,236 (65.2)* | 312/619 (50.4) | 1,458/2,168 (67.3)* | 250/417 (60.0) | 630/890 (70.8)* |
| **Antiaggregants, n/N (%)** | 2,025/8,408 (24.1) | 7,166/28,670  (25.0) | 2,515/3,809  (66.0)* | 4,381/6,891 (63.6) | 657/1,899 (34.6) | 2,177/6,144 (35.4) | 852/1,239 (68.8) | 1,593/2,363 (67.4) |
| **Anticoagulants, n/N (%)** | 1,565/8,408 (18.6)* | 4,598/28,670  (16.0) | 1,000/3,809 (26.3) | 1,708/6,891 (24.8) | 407/1,899 (21.4)* | 1,182/6,144 (19.2) | 328/1,239 (26.5) | 619/2,363 (26.2) |
| **ACEI or ARB, n/N (%)** | 2,457/8,408 (29.2) | 9,483/28,670 (33.1)* | 1,568/3,809 (41.2) | 2,962/6,891  (43.0) | 757/1,899 (39.9) | 2,614/6,144 (42.5)* | 613/1,239 (49.5) | 1,151/2,363 (48.7) |
| **Statins, n/N (%)** | 1,393/8,408 (16.6) | 5,700/28,670 (19.9)* | 1,986/3,809 (52.1)* | 2,909/6,891 (42.2) | 620/1,899 (32.6) | 2,175/6,144 (35.4)* | 736/1,239 (59.4)* | 1,245/2,363 (52.7) |

* denotes a p value <0.05 in the comparison between men and women.

SBP: Systolic blood pressure; DBP: Diastolic blood pressure; BMI: Body mass index; GFR: estimated glomerular filtration rate; ACEI: Angiotensin-converting enzyme inhibitors; ARB: Angiotensin receptor blockers.
