# Supplementary material for: All-cause mortality and cardiovascular events in a Spanish nonagenarian cohort according to type 2 diabetes mellitus status and established cardiovascular disease
Source: BMC Geriatr. 2022 Mar 18;22:224. doi: 10.1186/s12877-022-02893-z (PMC8931574; doi:10.1186/s12877-022-02893-z)
Supplement: Supplementary file 2 — Additional file 2. [file 12877_2022_2893_MOESM2_ESM.docx]

|  | **Group 1:**  **No T2DM, no prior CVD**  **(n= 33,091)** | | | **Group 2:**  **No T2DM, prior CVD**  **(n= 9,991)** | | | **Group 3:**  **T2DM, no prior CVD**  **(n= 7,470)** | | | **Group 4:**  **T2DM, prior CVD**  **(n= 3,382)** | | |
| --- | --- | --- | --- | --- | --- | --- | --- | --- | --- | --- | --- | --- |
|  | **Men**  **(n=7,316)** | **Women**  **(n=25,775)** | **p**  **value** | **Men**  **(n=3,527)** | **Women**  **(n=6,464)** | **p value** | **Men**  **(n=1,756)** | **Women**  **(n=5,714)** | **p**  **value** | **Men**  **(n=1,162)** | **Women**  **(n=2,220)** | **p value** |
| **All-cause mortality, n (%)** | 2,328 (31.8) | 6,855 (26.6) | <0.001 | 1,295 (36.7) | 2,090 (32.3) | <0.001 | 604 (34.4) | 1,714 (30.0) | <0.001 | 495 (42.6) | 865  (39.0) | 0.041 |
| **Non-fatal AMI, n (%)** | 36 (0.5) | 90 (0.3) | 0.080 | 11 (0.3) | 22 (0.3) | 0.813 | 8 (0.5) | 35 (0.6) | 0.447 | 13 (1.1) | 13 (0.6) | 0.092 |
| **Non-fatal stroke, n (%)** | 210 (2.9) | 693 (2.7) | 0.400 | 104 (2.9) | 217 (3.4) | 0.269 | 54 (3.1) | 174 (3.0) | 0.949 | 45 (3.9) | 87 (3.9) | 0.947 |
| **Heart failure, n (%)** | 343 (4.7) | 981 (3.8) | 0.001 | 194 (5.5) | 340 (5.3) | 0.609 | 80 (4.6) | 219 (3.8) | 0.176 | 76 (6.5) | 111(5.0) | 0.063 |
| **CPO1, n (%)** | 2,485 (34.0) | 7,360 (28.6) | <0.001 | 1,364 (38.7) | 2,226 (34.4) | <0.001 | 646 (36.8) | 1,857 (32.5) | 0.001 | 524 (45.1) | 921  (41.5) | 0.044 |
| **CPO2, n (%)** | 2,685 (36.7) | 7,947 (30.8) | <0.001 | 1,461 (41.4) | 2,412 (37.3) | <0.001 | 687 (39.1) | 1,987 (34.8) | 0.001 | 555 (47.8) | 975  (43.9) | 0.033 |

**Supplementary Table S2. Two-year cumulative incidence of all-cause mortality and cardiovascular events in the nonagenarian population studied, according to type 2 diabetes mellitus (T2DM) status, and the presence or absence of prior cardiovascular disease (CVD): differences between men and women.**

AMI: Acute myocardial infarction. CPO1: Composite primary outcome, number 1 (all-cause mortality, non-fatal myocardial infarction, non-fatal stroke). CPO2: Composite primary outcome, number 2 (all-cause mortality, non-fatal acute myocardial infarction, non-fatal stroke, heart failure).
