# Supplementary material for: All-cause mortality and cardiovascular events in a Spanish nonagenarian cohort according to type 2 diabetes mellitus status and established cardiovascular disease
Source: BMC Geriatr. 2022 Mar 18;22:224. doi: 10.1186/s12877-022-02893-z (PMC8931574; doi:10.1186/s12877-022-02893-z)
Supplement: Supplementary file 3 — Additional file 3. [file 12877_2022_2893_MOESM3_ESM.docx]

|  | **HR** | **CI 95%** | **p value** |
| --- | --- | --- | --- |
| **Age** | 0.99 | 0.94-1.05 | 0.75 |
| **Male Gender** | 1.25 | 0.94-1.67 | 0.13 |
| **Group 1: T2DM (-) & CVD (-)** | 1 |  |  |
| **Group 2: T2DM (-) & CVD (+)** | 0.85 | 0.58-1.25 | 0.41 |
| **Group 3: T2DM (+) & CVD (-)** | 1.51 | 1.06-2.13 | 0.02 |
| **Group 4: T2DM (+) & CVD (+)** | 2.00 | 1.31-3.08 | 0.01 |

**Supplementary Table 3. Adjusted effect on the incidence of 228 cases of acute myocardial infarction of the different categories according to type 2 diabetes mellitus (T2DM) status and presence or absence of prior cardiovascular disease.**

Adjusted by history of Chronic obstructive pulmonary disease, solid cancer, leukemia/lymphoma, chronic kidney disease, dementia, heart failure, deep vein thrombosis or pulmonary thromboembolism, and atrial fibrillation.
