# Supplementary material for: All-cause mortality and cardiovascular events in a Spanish nonagenarian cohort according to type 2 diabetes mellitus status and established cardiovascular disease
Source: BMC Geriatr. 2022 Mar 18;22:224. doi: 10.1186/s12877-022-02893-z (PMC8931574; doi:10.1186/s12877-022-02893-z)
Supplement: Supplementary file 4 — Additional file 4. [file 12877_2022_2893_MOESM4_ESM.docx]

|  | **HR** | **CI 95%** | **p value** |
| --- | --- | --- | --- |
| **Age** | 1.01 | 0.99-1.02 | 0.68 |
| **Male Gender** | 1.01 | 0.90-1.14 | 0.83 |
| **Group 1: T2DM (-) & CVD (-)** | 1 |  |  |
| **Group 2: T2DM (-) & CVD (+)** | 1.16 | 1.02-1.32 | 0.02 |
| **Group 3: T2DM (+) & CVD (-)** | 1.12 | 0.97-1.29 | 0.14 |
| **Group 4: T2DM (+) & CVD (+)** | 1.45 | 1.20-1.74 | <0.01 |

**Supplementary Table 4. Adjusted effect on the incidence of 1,584 cases of stroke of the different categories according to type 2 diabetes mellitus (T2DM) status and presence or absence of prior cardiovascular disease.**

Adjusted by history of Chronic obstructive pulmonary disease, solid cancer, leukemia/lymphoma, chronic kidney disease, dementia, heart failure, deep vein thrombosis or pulmonary thromboembolism, and atrial fibrillation.
